# Exploring the Genetic Landscape of Vitiligo in the Pura Raza Español Horse: A Genomic Perspective

**DOI:** 10.3390/ani14162420

**Published:** 2024-08-21

**Authors:** Nora Laseca, Antonio Molina, Davinia Perdomo-González, Chiraz Ziadi, Pedro J. Azor, Mercedes Valera

**Affiliations:** 1 Departamento de Agronomía, Escuela Técnica Superior de Ingeniería Agronómica, Universidad de Sevilla, Ctra. Utrera Km 1, 41013 Sevilla, Spain; noralaseca@gmail.com (N.L.); davpergon1@alum.us.es (D.P.-G.); 2 Real Asociación Nacional de Criadores de Caballos de Pura Raza Española (ANCCE), Cortijo de Cuarto (Viejo), 41014 Sevilla, Spain; pedroazor@lgancce.com; 3 Departamento de Genética, Universidad de Córdoba, Ctra. Madrid Km 396, 44014 Córdoba, Spain; ge1moala@uco.es (A.M.); z72zizic@uco.es (C.Z.)

**Keywords:** depigmentation disorder, GWAS, horse, single-step genomic BLUP, candidate genes, skin diseases

## Abstract

**Simple Summary:**

Recent advances in equine genomics have opened up new avenues for understanding complex traits such as vitiligo, a chronic, long-lasting autoimmune disorder, which, in horses, causes some areas of the skin to lose their natural colour. To identify genomic regions associated with vitiligo depigmentation and susceptibility in equine populations, we performed a genome-wide association study for this disorder in Pura Raza Español horses. Using the weighted single step genomic best linear unbiased prediction (wssGBLUP) analysis methodology for the first time in an equine species, a total of 10 significant genomic regions were associated with vitiligo in different areas. Some of the significant genomic regions were shared among different vitiligo traits and explained most of the additive variance. The importance of these studies lies in their potential to unravel the underlying genetic architecture of vitiligo in horses, shedding light on the molecular mechanisms involved in the development of this disease in this species. Furthermore, it allows us to initiate selective breeding strategies aimed at reducing the prevalence of vitiligo in horses.

**Abstract:**

Vitiligo is a depigmentation autoimmune disorder characterized by the progressive loss of melanocytes leading to the appearance of patchy depigmentation of the skin. The presence of vitiligo in horses is greater in those with grey coats. The aim of this study was therefore to perform a genome-wide association study (GWAS) to identify genomic regions and putative candidate loci associated with vitiligo depigmentation and susceptibility in the Pura Raza Español population. For this purpose, we performed a wssGBLUP (weighted single step genomic best linear unbiased prediction) using data from a total of 2359 animals genotyped with Affymetrix Axiom™ Equine 670 K and 1346 with Equine GeneSeek Genomic Profiler™ (GGP) Array V5. A total of 60,136 SNPs (single nucleotide polymorphisms) present on the 32 chromosomes from the consensus dataset after quality control were employed for the analysis. Vitiligo-like depigmentation was phenotyped by visual inspection of the different affected areas (eyes, mouth, nostrils) and was classified into nine categories with three degrees of severity (absent, slight, and severe). We identified one significant genomic region for vitiligo around the eyes, eight significant genomic regions for vitiligo around the mouth, and seven significant genomic regions for vitiligo around the nostrils, which explained the highest percentage of variance. These significant genomic regions contained candidate genes related to melanocytes, skin, immune system, tumour suppression, metastasis, and cutaneous carcinoma. These findings enable us to implement selective breeding strategies to decrease the incidence of vitiligo and to elucidate the genetic architecture underlying vitiligo in horses as well as the molecular mechanisms involved in the disease’s development. However, further studies are needed to better understand this skin disorder in horses.

## 1. Introduction

Vitiligo is a multifactorial depigmentation disorder characterized by the progressive loss of the melanocytes which produce the epidermal pigment. This autoimmune disorder leads to the appearance of depigmented patches on the skin and hair [1]. The pathogenesis of vitiligo is explained by the interplay of intrinsic melanocyte defects, autoimmune responses, and genetic and environmental influences [2]. In vitiligo, skin melanocytes are partially or completely lost, and no melanin is synthesized in this area. However, the cause of the destruction of epidermal or follicular melanocytes is complex and not yet fully understood. In humans, vitiligo is the most common depigmenting disorder, with an estimated prevalence of approximately 0.5–2.0% of the world’s population [3]. Vitiligo affects not only humans, but also various animal species [4], including horses [5].

In equine species, vitiligo often manifests as patchy depigmentation of the skin around the eyes, muzzle, and the perianal and genitalia region, which can expand over time. This skin disorder occurs predominantly in animals with a grey coat colour, such as Pura Raza Española (PRE) horses and Lipizzan horses [6,7], and reaches a prevalence of about 80% in horses of 15 years of age or older [8]. The defect, while not life threatening, can have a significant impact on the aesthetic value of the animal.

The genetic basis of vitiligo in humans has been partially elucidated through genome-wide association studies (GWASs), which have identified several loci and candidate genes associated with the disorder [9,10,11,12,13,14]. However, there are very few studies on understanding the genetic basis of vitiligo in horses. Existing studies on equine vitiligo are limited, with most focusing on clinical presentation and histopathological features [15] rather than genetic factors. Despite this, some studies have suggested there may be a hereditary component to the disorder in horses [6,8,16].

Genomic studies in equines have advanced considerably in recent years, offering new opportunities to explore the genetic basis of various disorders, including vitiligo. Whole-genome sequencing and high-throughput genotyping have enabled us to identify genetic variants associated with complex traits in horses. However, there are very few comprehensive genome-wide association analyses specifically targeting vitiligo in horses [7]. In this context, the GWAS shows potential for clarifying the molecular mechanisms underlying vitiligo and for identifying the genetic markers that could facilitate early diagnosis and management.

The Pura Raza Español is one of the most widespread equine breeds, with more than 26.23% of its census (275,018 active animals) distributed over 67 different countries around the world. The PRE studbook accepts animals with a diversity of coat colour patterns. Its predominant coat is grey (55.14%), followed by other coats such as bay (32.46%), black (9.72%), chestnut (2.2%), and dilutions of these basic coats (0.49) [17]. Due to its predominantly grey colouring, the PRE is particularly prone to vitiligo, as already demonstrated [6,7,8], the prevalence of vitiligo was higher in grey coats such as those of the PRE. Since 2012, it has been standard practice to record a vitiligo score in the breed registers of the PRE. In order to be registered in the studbook, for which the Royal National Association of Spanish Purebred Horse (ANCCE, Seville, Spain) is responsible, PRE horses undergo a morphological evaluation, which includes zoometric variables and linear variables with a score of 9, including the evaluation of vitiligo at the level of the eyes, nostrils, and mouth (ANCCE, Seville, Spain 2024). These morphological data are subsequently used for the genetic evaluation that is systematically carried out on the breed in its breeding programme. This study aims to identify the genomic regions associated with vitiligo in PRE horses using a comprehensive genome-wide association analysis. To achieve this, by leveraging high-throughput genotyping technologies, we seek to reveal the genetic architecture of vitiligo in this breed and provide insights into the potential hereditary factors contributing to the disorder. The identification of genomic regions could improve diagnostic tools, management practices, and potentially, genomic selection for vitiligo in horses.

## 2. Materials and Methods

### 2.1. Animals and Phenotypes

**Figure 1:**
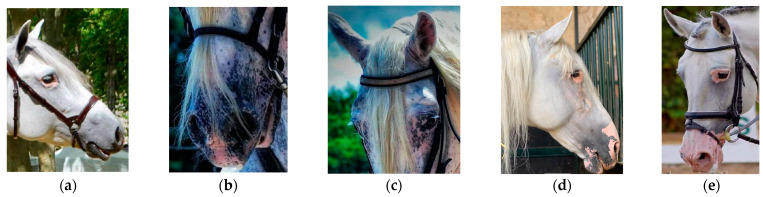
Phenotypic levels of vitiligo depigmentation in Pura Raza Español horses. (**a**) Slight degree in eyes; (**b**) slight degree in mouth and nostrils; (**c**) slight degree in eyes; (**d**) severe degree in eyes, mouth, and nostrils; and (**e**) severe degree in eyes, mouth, and nostrils. All the animals have grey coats.

Phenotypic data for vitiligo were collected for a total of 56,643 animals (18,855 males and 38,341 females) during the mandatory morphological assessment, which occurs once in a horse’s lifetime before they are officially registered in the main section of the PRE studbook. The evaluations were conducted by a total of 12 specially trained veterinarians who were responsible for performing standardized aptitude tests in this breed. These controls are carried out following the procedures outlined by Sánchez-Guerrero et al. [6], in which a series of traits are recorded by experienced judges as linear morphological classification, ranging from 1 (complete absence of the defect) to 9 (severe defect) (Table 1 and Figure 1). The traits measured were vitiligo around eyes (VE), vitiligo around mouth (VM), and vitiligo around nostrils (VN). A relationship matrix was generated with 109,981 animals (32,697 males and 77,284 females) and contained a maximum of 24 generations with an average of 5.7 complete generations.

### 2.2. Genotyping and Quality Control

Genomic DNA was provided by the ANCCE Molecular Genetics Laboratory from different biological samples (blood and hair). The most representative horses of the population were selected for genotyping, belonging to more than 600 studs and with a low average relatedness among individuals. These horses were genotyped using the high density Affymetrix Axiom™ Equine 670 K SNP (single nucleotide polymorphism) (Thermofisher, Waltham, MA, USA) (n = 2359) and the medium density GGP (GeneSeek Genomic Profiler™, San Diego, CA, USA) Equine Array V5 (Neogen, Lansing, MI, USA) (n = 1763) genotyping arrays. The raw genotype data were analyzed following the “Best Genotyping Practices Workflow” using the Axiom Analysis Suite 5.0 software with the default parameters (Dish Quality Control > 0.82 and call rate > 0.97) and a bioinformatics pipeline including an initial variant call and genotype curation using GenomeStudio Software 2.0.5 and PLINK v1.9 software [18]. Next, the genotypes of the common markers of both technologies were merged and quality control analyses were performed using the PLINK v1.9 software. SNP markers with a call rate below 95% were discarded from further analysis. The final genomic data included 60,136 SNPs distributed on all 32 chromosomes.

### 2.3. Weighted Single-Step Genome-Wide Association Analysis

A weighted single-step genomic best linear unbiased prediction (wssGBLUP) approach was used to analyze VE, VM, and VN. The variance components were estimated using the REML methodology, and the results were then used to predict the genomic estimated breeding values (GEBVs). The multivariate linear model of analysis used for the studied traits was as follows:

(1) y=Xb+Za+Wp+e

where y is the vector of phenotypic observations for the corresponding trait; b is the vector of fixed effects, including inbreeding as a linear covariate, sex (two levels: male and female), age assessment (2 levels: young < 4 years old and adult ≥ 4 years old), coat colour (4 levels: bay, chestnut, black and grey), and geographical area (4 levels: Spain, rest of Europe, North America and Mexico, and South and Central America); a is the vector of random additive genetic effects; *p* is the vector of random permanent environmental effects; e is the vector of random residuals; and X, Z, and W are the incidence matrices of b, a, and *p*, respectively.

It was assumed that $a\sim N(0,H\sigma_{a}^{2}$), $p\;\sim \;N(0,I\sigma_{p}^{2})$, and $e\;\sim \;N\left(0,\;I\sigma_{e}^{2}\right)$, where $\sigma_{a}^{2}$, $\sigma_{p}^{2}$, and $\sigma_{e}^{2}$ are the additive genetic, permanent environmental, and residual variances, respectively. For all the traits, H is the matrix that combines the numerator relationship matrix (A) with the genomic relationship matrix (G), as described by Aguilar et al. [19], and I is an identity matrix. The inverse of matrix H is:

(2) $$H^{-1}=A^{-1}+\left[\begin{array}{cc} 0 & 0\\ 0 & G^{-1}-A_{22}^{-1} \end{array}\right]$$

where A is the pedigree-based relationship matrix for all animals; A_22_ is the pedigree-based relationship matrix for genotyped animals; and G is the genomic relationship matrix for genotyped animals, obtained by the following procedure [20]:

(3) $$G=\frac{Z{DZ}^{\prime }}{\sum_{i=1}^{N}2\hat{p_{i}}\left(1-\hat{p_{i}}\right)}$$

where Z’ is a matrix of gene content adjusted for allele frequencies (0, 1, or 2 for aa, Aa, and AA, respectively); D is a diagonal matrix of weights for SNP variances (initially, D = I); N is the number of SNPs; and p_i_ is the minor allele frequency of i-th SNP.

Next, we obtained the estimates of SNP effects ($\hat{g}$) by back-solving GEBVs according to Wang et al. [21], as follows:

(4) $$\hat{g}=Z^{\prime }{\left(Z^{\prime }Z\right)}^{-1}\hat{u}$$

After predicting the SNP effects, we gave weights to the markers based on the SNP solutions. The weights of the SNPs were included in all the analyses, which meant that the effect of the SNPs and the effect of the animals had to be recalculated.

The percentage of genetic variance explained by the i-th set of SNPs included in a 1 Mb window (i-th SNP window) was calculated as described by Wang et al. [21]:

(5) $$\frac{Var(a_{i})}{\sigma_{a}^{2}}\;\times \;100\%=\frac{Var\;(\sum_{j=1}^{x}Z_{j}{\hat{u}}_{j})}{\sigma_{a}^{2}}\;\times \;100\%$$

where $a_{i}$ is the genetic value of the i-th SNP window that consists of a region of consecutive SNPs located within 1 Mb, $\sigma_{a}^{2}$ is the total additive genetic variance, $Z_{j}$ is the vector of gene content of the j-th SNP for all individuals, and ${\hat{u}}_{j}$ is the effect of the j-th SNP within the i-th window.

We used the BLUPF90+ software [22] for the wssGBLUP to estimate genetic parameters of the studied traits. We then performed the GWAS analysis with the POSTGSF90 program [19] using the 1 Mb overlapping windows option and selected the SNPs with additive genetic variance explained above 1%. Manhattan plots were then created using the R software 4.4.1.

### 2.4. Functional Analysis of Significant Genomic Regions

To identify potential candidate genes associated with vitiligo, the significant genomic regions were used to perform a functional enrichment analysis. Genes were annotated within genomic regions using the software Ensembl BioMart tool [23] with the *Equus caballus* reference genome (EquCab3.0. http://www.ensembl.org/Equus_caballus/Info/Index (accessed on 12 August 2024)). The function of the candidate genes, and their putative relationship with the disorder, as well as the metabolic pathways and biological processes involved in vitiligo, were established by analysis using the DAVID (Database for Annotation Visualization and Integrated Discovery) software (v2024q2) [24] and AmiGO 2 (Gene Ontology) software [25] and the literature available in public databases.

## 3. Results

### 3.1. Descriptive Statistics

The descriptive statistics of the phenotypes of vitiligo are shown in Table 2, which also shows the prevalence of vitiligo analyzed in the PRE population. Most of the animals (>79%) were included in the group of unaffected animals for each area analyzed. Notably, the area with the highest prevalence of vitiligo was the mouth (20.94%), followed by the nostrils (17.53%), with the eyes being the area with the lowest prevalence of vitiligo (3.99%) in the PRE population.

The variance components, SNP-based heritability coefficients, and genetic correlations are shown in Table 3. SNP-based heritability estimates were moderate for VN and lower for VE and VM, with a range from 0.13 (s.d. ± 0.006) for vitiligo around the mouth to 0.28 (s.d. ± 0.009) for vitiligo around the nostrils.

The highest genetic correlation was found between vitiligo around the mouth and the nostrils (0.79 (s.d. ± 0.017)). Meanwhile, the genetic correlations of vitiligo around the eyes with vitiligo around the mouth and nostrils were similar (0.55 (s.d. ± 0.028) and 0.52 (s.d. ± 0.023), respectively).

Table 4 shows the range of solutions for the fixed effects (sex, age, coat colour, and geographical area) and covariate effects (inbreeding) affecting vitiligo traits. It should be noted that all these effects, with their different degrees, had a significant effect on vitiligo parameters, to a greater or lesser extent.

### 3.2. Genome-Wide Association Studies

**Figure 2:**
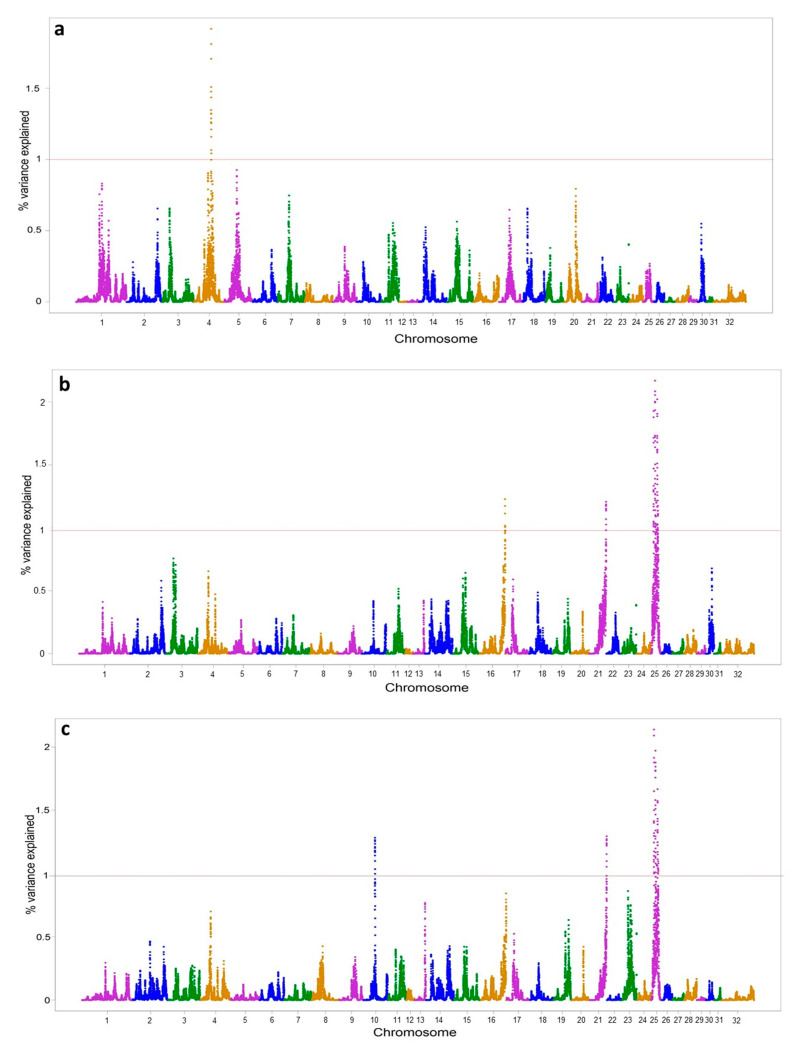
Manhattan plot of SNP additive genetic variance (y axis) explained at each of the SNPs by 1 Mb window of adjacent SNPs for traits (**a**) VE: vitiligo around eyes; (**b**) VM: vitiligo around mouth; (**c**) VN: vitiligo around nostrils. Red line: percentage of additive genetic variance above 1%.

The genomic regions which explained an additive genetic variance above 1% were selected. The genome-wide association analyses resulted in the detection of 10 significant genomic regions on 5 chromosomes (ECA) associated with vitiligo (Figure 2 and Table 5). For VE, one significant genomic region was detected on ECA4; for VM, eight significant genomic regions were identified on ECA16, ECA21, and ECA25; and for VN, seven significant genomic regions were detected on ECA10, ECA21, and ECA25. The phenotypes VN and VM shared six significant genomic regions on chromosomes 21 and 25. All in all, we found a total of 78 genes in genomic regions with more than 1% of variance explained for the vitiligo trait, 18 of which were related to the immune system, melanocytes, tumour suppression, metastasis, cutaneous carcinoma, and skin.

## 4. Discussion

Interest in exploring genomic regions associated with diseases such as vitiligo has increased in recent years, as has the growing number of genotyped animals. Genome-wide association analysis (GWAS) provides an effective method for identifying potential genetic variations and candidate genes in domestic animals, particularly for traits with a significant economic value [26]. As a result, the GWAS was performed to discover regions associated with vitiligo, which can help to initiate breeding strategies aimed at reducing the prevalence of this disease. Most studies on vitiligo have been conducted on grey-coated horses, as it was observed that this coat colour had the highest prevalence of vitiligo [6,8,16]. However, our study was conducted on all the major coat colours (bay, chestnut, black, and grey) of the Pura Raza Español horse because there is some evidence from other studies that vitiligo may be linked to other coat colours.

### 4.1. Prevalence and Genetics Parameters

The overall prevalence of vitiligo found was 20.94% around the mouth, followed by 17.53% around the nostrils. These results were similar to those reported in other breeds such as the Lipizzan horse (21.7%) [7]. However, vitiligo around the eyes had a lower prevalence (3.99%), as previously reported in another study of PRE horse [6].

In this work, inbreeding was significantly associated with the presence of vitiligo, as it has been in the PRE with the presence of white facial markings [27], hair whorls [28] or melanomas [6]. Sex also influenced vitiligo, with the occurrence of vitiligo (except for VM) higher in females than in males, as did the geographical area (Table 4). These results were consistent with previous findings on vitiligo in the PRE population [6]. Our study also confirmed the effect of age and coat colour on the prevalence of vitiligo in horses, as previously described in other studies (in Grey horses [8], in Old Kladruber horses [16], and in PRE [6]). In this sense, one limitation of this study is that the prevalence of the disease is influenced by coat colour, and the frequency of specific coat types can vary by geographical region. This breed is present in more than 65 countries, each with different preferences for coat colours. In this analysis, sampling was conducted in Spain, where the distribution of coat colours may differ from that in other countries, potentially affecting the observed disease prevalence. Additionally, among the genotyped animals, none had dilute coats, reflecting the low prevalence of this coat type in the PRE population.

The SNP-based heritability estimate for depigmentation in eyes was 0.17 ± 0.007, which is higher than the estimated heritability in the PRE without considering genomics, with values of 0.09 ± 0.019 [6]. In the case of vitiligo around the mouth and nostrils, it is difficult to accurately compare our results with others reported [6,16], since other studies combined mouth and nostril vitiligo traits into the same trait (facial vitiligo), with *h*^2^ values of 0.34 ± 0.06 and 0.44 ± 0.031, respectively. If the heritability of vitiligo around the nostrils alone (0.28 ± 0.009) is taken into account, the *h^2^* is similar to that globally estimated for the Lipizzan horse (0.31 ± 0.13) [7] with genomics. Interestingly, our heritability results, which showed acceptable values, improved further when estimated with genomic data, which suggests that the incorporation of genomics improves the accuracy of selection. Nevertheless, their magnitude leads us to believe that selective breeding to avoid one vitiligo trait may effectively reduce these traits in the PRE population.

The medium-high correlation indicates that the molecular mechanisms that determine this disease are the same globally, albeit with differences in some genes or their expression. This fact also coincides with the existence of different candidate genes for each area analyzed, along with other common ones. In any case, the magnitude means that the selection of animals with greater resistance for a specific area presented a response to positive correlated selection in the other two areas (especially between VM and VN). In a previous study in this breed, the r_g_ between vitiligo around the eyes and a combination of vitiligo traits around the mouth and nostrils was 0.42 ± 0.084 [6].

### 4.2. Genomic Association with Vitiligo

In the GWAS study on vitiligo depigmentation in PRE horses, we identified a total of 10 genomic regions (in chromosomes 4, 10, 16, 21, and 25), which explain a large proportion of the additive genetic variance, with six genomic regions on chromosome 25 identified for two of the traits studied (VM and VN). This suggests that these regions identified on ECA25 may be hotspots for vitiligo. In addition, the genomic regions associated with the highest proportion of additive genetic variation were also located on this chromosome. In 9 of the 10 significant regions, we found genes related to skin, melanocytes, tumour suppression, metastasis, cutaneous carcinoma, and the immune system. These findings are in accordance with the nature of the disease, as it is a complex (polygenic and multifactorial) disease, so multiple genes are involved, as well as a combination of genetic and environmental factors that contribute to the development of this disease.

In region 4: 62,791,008–63,734,743 pb, associated with vitiligo eyes, we identified two genes, the *BBS9* (Bardet-Biedl syndrome 9) gene and the *PR9* (PR9 pre-mRNA splicing factor) gene. McCreery et al. [29] reported that mutations in the *BBS9* gene were involved in the evolution of metastatic tumours in a study of a classical skin tumour model in a genetically heterogeneous mouse population using whole exome sequencing of multiple lesions. Meanwhile, mutations in pre-mRNA processing factors (PR9) have been linked to 15–20% of cases of autosomal dominant retinitis pigmentosa (the most common heritable retinal disease in humans and characterized by progressive degeneration of the photoreceptors and/or retinal pigment epithelium, leading to blindness) [30].

The *ZNF292* and *AKIRIN2* genes were identified on ECA10 in the region 40,539,712–41,516,383 pb. The zinc finger protein 292 (*ZNF292*) gene was reported to be a target gene regulated by *HIF1α* in melanocytes whose gene expression change under hypoxia and was associated with primary human melanoma tumours [31]. Also, the *AKIRIN2* (akirin 2) gene was shown to play an important role in immune responses and tumorigenesis [32]. Another gene related to the immune system was found on chromosome 16 (87,269,741–88,241,572 pb). This gene, *DHX36*, may play an important role in DNA-mediated innate immunity [33].

Interestingly, in the 57,490,258–58,411,259 pb region of chromosome 21 associated with two traits (VM and VN), we found five candidate genes (*LPCAT1*, *CLPTM1L*, *TERT*, *TRIP13*, and *BRD9*), all of which were related to the main types of skin cancer, i.e., melanoma and cutaneous squamous cell carcinoma. Several clinical observations [34] suggest a relationship between vitiligo and malignant melanoma; in both diseases, antibodies develop against similar antigens present in normal melanocytes and in malignant melanoma cells. The lysophosphatidylcholine acyltransferase 1 (*LPCAT1*) gene was identified as a cancer promoter in cutaneous squamous cell carcinoma [35]. The *CLPTM1L* gene encodes a membrane protein whose overexpression in cisplatin-sensitive cells causes apoptosis, and polymorphisms at this locus have been reported to increase susceptibility to many cancer types, including melanoma [36]. Telomerase reverse transcriptase (*TERT*) mutations are key players in the genesis and progression of melanoma. Mutations in the *TERT* promoter region are associated with reduced disease-free survival, increased tumour recurrence, and a higher rate of metastasis in melanoma: in other words, these mutations increase *TERT* expression, which helps cancer cells grow and survive [37]. In addition, Lu et al. [38] revealed that the *TRIP13* (thyroid hormone receptor interactor 13) gene is a novel prognostic biomarker and a potential therapeutic target for melanoma treatment. Finally, it was recently reported that the bromodomain-containing 9 (*BRD9*) gene is over-expressed in melanoma and that a high expression of *BRD9* correlates with poorer survival, suggesting that it may be a therapeutic target [39]. There is also evidence that *BRD9* is potentially a tumour suppressor in both uveal and cutaneous melanoma [40].

Regarding ECA25, six significant genomic regions were identified, although genes related to biological processes associated with vitiligo (*TXN*, *LPAR1*, *SLC46A2*, *PRPF4*, *TRIM32*, *STOM*, *BRINP1*, and *DAB2IP*) were only found in five of them. Among these genes are several related to the skin barrier, such as *TXN* (thioredoxin), which is involved in cell defences against oxidative damage on proteins and in maintaining the redox status in the cell [41], and the *LPAR1* gene (lysophosphatidic acid receptor 1), which has been identified as a critical axis for melanoma invasion [42]. Another study reported that *LPA/LPAR1* signalling induces keratinocyte hyperproliferation by promoting cell cycle progression, contributing to the pathogenesis of chronic inflammatory skin disease [43]. The solute carrier family 46 member 2 gene (*SLC46A2*), which is highly expressed in mammalian epidermal keratinocytes, was shown to be essential for the transport of NOD1-activating mucopeptides, with critical functions in the skin barrier, and was identified as an important target for anti-inflammatory interventions [44]. Genes related to melanin production were also found, such as the pre-mRNA processing factor 4B (*PRP4*). Ahmed et al. [45] suggested that the *PRP4* gene inhibits melanin production in murine melanoma cells, blocks Ca2+ influx through CaSR desensitization, and modulates the actin cytoskeleton through down-regulation of the AC-cAMP pathway; together, these observations collectively promote cutaneous carcinogenesis. Among other genes related to the immune system are *TRIM32* or *STOM*, and altered innate immunity is a feature of certain inflammatory skin diseases. Liu et al. [46] demonstrated that deficiency in *TRIM32* (a member of the tripartite motif (TRIM) protein family found to regulate host inflammatory responses) contributes to a T-helper type 2 biased response and predisposes mice to inflammatory skin disease traits. Meanwhile, Ono et al. [47] linked *STOM* expression to Th1 cells (which play an important role in the pathogenesis of human inflammatory, allergic, and autoimmune diseases) and suggested that these molecules are new markers of Th1 cells. Finally, the *BRINP1* (BMP/retinoic acid inducible neural specific 1) gene is associated with hair-greying processes in humans [48], while the *DAB2IP* (DAB2 interacting protein) gene has been reported to play a role in tumour suppression and metastasis [49].

## 5. Conclusions

Our results, using a weighted single-step GBLUP, have shown that the vitiligo trait has moderate heritability, which ensures a response to selection if used as a criterion for choosing animals less prone to developing the disease. The genetic correlation for disease propensity in the eyes, mouth, and nostrils is medium-high, indicating similar molecular mechanisms, as confirmed by the GWAS analyses, which identified genomic areas with a high impact common to these areas and specific to some areas. We identified 10 genomic regions associated with vitiligo in the three affected areas, involving 18 genes linked to biological processes such as the immune system, melanocytes, skin, tumour suppression, and melanoma. These findings will help us to implement selective breeding strategies to reduce the incidence of vitiligo in horses and provide additional knowledge on genomic variation and the candidate genes involved in the genetic mechanisms of vitiligo and the molecular mechanisms involved in the disease’s development. However, further studies are required to better understand this depigmentation skin disorder in horses.

## Figures and Tables

**Table 1:** Classification of degrees of vitiligo in Pura Raza Español horses.

Score	1	2	3	4	5	6	7	8	9
Categories	Absent	*	Few	*	Some	*	Many	*	Widespread
Degrees	Absent	Slight (2 ≤ score ≥ 5)				Severe (score ≥ 6)			

* Intermediate value between adjacent categories.

**Table 2:** Descriptive statistics and prevalence of vitiligo in the Pura Raza Español population. SNP-based heritability estimates of vitiligo traits around eyes, mouth, and nostrils.

Area	N	Descriptive Statistics		Prevalence (%)		
		Mean (s.d.)	Range	Absent	Slight	Severe
Eyes	56,641	1.16 (0.908)	1–9	96.01	2.92	1.07
Mouth	20,425	1.69 (1.565)	1–9	79.07	16.99	3.95
Nostrils	56,632	1.57 (1.475)	1–9	82.47	13.60	3.93

N: horses with phenotype; s.d.: standard deviation.

**Table 3:** Estimations of variance components, heritability, and genetic correlations of vitiligo in Pura Raza Español population using a multivariate wssGBLUP.

Parameter	Trait		
	VE	VM	VN
$\sigma_{a}^{2}$ (s.d.)	0.13 (0.006)	0.13 (0.006)	0.61 (0.022)
$\sigma_{e}^{2}$ (s.d.)	0.69 (0.006)	0.83 (0.006)	1.55 (0.017)
$h^{2}$ (s.d.)	0.17 (0.007)	0.13 (0.006)	0.28 (0.009)
**rg** (s.d.)	**VE–VM** 0.55 (0.028)	**VE–VN** 0.52 (0.023)	**VM–VN** 0.79 (0.017)

$\sigma_{a}^{2}$: additive genetic variance; $\sigma_{e}^{2}$: residual variance; $h^{2}:$ heritability; rg: genetic correlation; s.d.: standard deviation; VE: vitiligo around eyes; VM: vitiligo around mouth; VN: vitiligo around nostrils.

**Table 4:** Solutions (standard error) of wssBLUP models for fixed effects and covariate effects to vitiligo in the Pura Raza Español population analyzed.

Effect	Levels	VE	VM	VN
**Inbreeding**	b	0.54 (0.101)	0.73 (0.107)	1.45 (0.163)
**Sex**	1	1.31 (0.089)	1.28 (0.085)	1.79 (0.179)
	2	1.32 (0.089)	1.27 (0.085)	1.86 (0.179)
**Age**	1	0.10 (0.008)	0.15 (0.008)	0.24 (0.012)
	2	<0.00 (0.00)	<0.00 (0.00)	<0.00 (0.00)
**Coat colour**	1	−0.10 (0.018)	0.13 (0.019)	−0.07 (0.029)
	2	−0.21 (0.017)	−0.13 (0.018)	−0.49 (0.027)
	3	−0.12 (0.020)	−0.11 (0.021)	−0.48 (0.033)
	4	<0.00 (0.00)	<0.00 (0.00)	<0.00 (0.00)
**Geographic area**	1	−0.09 (0.019)	<−0.00 (0.020)	−0.12 (0.032)
	2	−0.03 (0.022)	0.06 (0.023)	−0.07 (0.037)
	3	0.01 (0.021)	−0.03 (0.022)	−0.08 (0.035)
	4	<0.00 (0.00)	<0.00 (0.00)	<0.00 (0.00)

VE: vitiligo around eyes; VM: vitiligo around mouth; VN: vitiligo around nostrils.

**Table 5:** Genomic regions associated with vitiligo in Pura Raza Español population that explained a percentage of variance above 1%.

Chr.	Genomic Region (pb)	v.e. (%)	Trait	Candidate Genes
4	62,791,008–63,734,743	1.92	VE	*PDE1C*; *AVL9*; *KBTBD2*; *FKBP9*; *NT5C3A*; ***RP9***; ***BBS9***
10	40,539,712–41,516,383	1.28	VN	*HTR1E*; *CGA*; ***ZNF292***; *GJB7*; *SMIM8*; *C10H6orf163*; *CFAP206*; *RARS2*; *SLC35A1*; *ORC3*; ***AKIRIN2***
16	87,269,741–88,241,572	1.23	VM	*ARHGEF26*; ***DHX36***; *GPR149*
21	57,490,258–58,411,259	1.30	VM and VN	*IRX4*; *NDUFS6*; *MRPL36*; ***LPCAT1***; *SLC6A3*; ***CLPTM1L***; ***TERT***; *SLC6A18*; *SLC6A19*; *SLC12A7*; *NKD2*; ***TRIP13***; ***BRD9***
25	10,231,120–11,217,990	2.14	VM and VN	*TOPORSL*; *OR13F8*; *OR13D2H*; *OR13C2*; *OR13D2*; *OR13D2G*; *OR13C7O*; *OR13C8*; *OR13D1*; *NIPSNAP3A*; *NIPSNAP3B*; *ABCA1*
25	15,753,928–16,753,198	2.17	VM and VN	*PALM2AKAP2*; *C25H9orf152*; ***TXN***; *TXNDC8*; *SVEP1*; *MUSK*; ***LPAR1***; *OR2K2*; *ECPAS*
25	17,727,314–18,708,795	1.09	VM	*SNX30*; ***SLC46A2***; *SLC31A2*; *FKBP15*; *SLC31A1*; *CDC26*; ***PRPF4***; *RNF183*; *WDR31*; *BSPRY*; *HDHD3*; *ALAD*
25	20,520,115–21,509,611	1.20	VM and VN	*PAPPA*; *ASTN2*; ***TRIM32***
25	23,519,210–24,493,448	2.02	VM and VN	***BRINP1***
25	25,114,122–26,095,565	1.22	VM and VN	*C5*; *CNTRL*; *RAB14*; *GSN*; ***STOM***; ***DAB2IP***; *TTLL11*

Chr.: chromosome; pb: pair base; v.e.: variance explained; VE: vitiligo around eyes; VM: vitiligo around mouth; VN: vitiligo around nostrils. Genes related to biological processes associated with vitiligo are marked in bold.

## Data Availability

The data that support the findings of this study are available from the Real Asociación Nacional de Criadores de Caballos de Pura Raza Española (ANCCE). The data are available from the authors with the permission of ANCCE.
